# Investigation of a pertussis outbreak and comparison of two acellular booster pertussis vaccines in a junior school in South East England, 2019

**DOI:** 10.2807/1560-7917.ES.2021.26.12.2000244

**Published:** 2021-03-25

**Authors:** Elise Tessier, Helen Campbell, Sonia Ribeiro, Nick Andrews, Julia Stowe, Margot Nicholls, Jaime Morgan, David Litt, Norman K Fry, Gayatri Amirthalingam

**Affiliations:** 1 Immunisation and Countermeasures Division, National Infection Service, Public Health England, London, United Kingdom; 2 Surrey and Sussex Health Protection Team (South East), Public Health England, Surrey, United Kingdom; 3 Vaccine Preventable Bacteria Section, National Infection Service, Public Health England, London, United Kingdom

**Keywords:** pertussis, outbreak, acellular vaccine, 3-component, 5-component

## Abstract

In March 2019, a pertussis outbreak occurred in children in a junior school (7–11 years) in England who had been offered pertussis-containing booster vaccine at 40 months of age. In a case–control investigation, we assessed the extent of transmission and any difference in protection afforded to those who had previously received a booster 3- or 5-component acellular pertussis vaccine (aP). We took oral fluid specimens from the students to determine IgG antibodies against pertussis toxin (anti-PT). Parents of students attending the school were sent a questionnaire on pertussis symptoms and vaccination status was retrieved from general practitioner records for all students. Of 381 students, 134 (35.2%) were classified as pertussis cases, 133 by demonstration of significant anti-PT IgG titres and one clinically. There was no significant difference in the risk of pertussis between students receiving 3-component (33.7%) or 5-component (32.3%) aP boosters. However, pertussis infection differed significantly in school year 4, with 22.9%, 50.0%, 23.7% and 38.1% pertussis cases in years 3, 4, 5 and 6, respectively. The proportion of students with incomplete vaccinations recorded was higher than the proportion of those not covered according to the national reported coverage, possibly contributing to sustained transmission within the school.

## Introduction

Whooping cough (pertussis) is a highly contagious bacterial infection caused by a Gram-negative bacterium *Bordetella pertussis* that is transmitted by aerosol droplets. Pertussis symptoms typically last up to 3 months and include low-grade fever, coughing with ‘whooping’ sound in some infants and vomiting [1,2]. Globally, there are an estimated 50 million cases of pertussis annually with the highest incidence rate and a high number of pertussis-related deaths in infants younger than 4 months [1]. Vaccine schedules vary between countries including the number and type of pertussis-containing vaccines used. In an attempt to improve the control of pertussis, a number of countries have included additional booster doses beyond preschool [3]. However, despite offering booster vaccinations before school entry and in adolescence, outbreaks in primary schools have been noted in such countries, including China, Germany and the United States [4-6].

In England, as in the rest of the United Kingdom (UK), the routine immunisation programme consists of three primary infant doses of a pertussis-containing vaccine at 8, 12 and 16 weeks of age, and one preschool booster dose at 40 months (3 years and 4 months) of age [7,8]. Vaccines for the national immunisation programme are centrally procured and distributed across the country to general practitioner (GP) practices. As a result, specific age cohorts within the population will have received the same vaccine product, although during some periods, two different products were available for the pertussis programme at the same time. This centralised procurement does provide a unique opportunity to evaluate and compare effectiveness of different vaccine products.

In 1990, an accelerated diphtheria, tetanus toxoids and whole-cell pertussis (DTwP) schedule was introduced to improve protection earlier in infancy, where the risk of severe disease is highest. The primary infant schedule changed from a whole-cell pertussis vaccine (wP) to a 5-component acellular pertussis vaccine (aP), Pediacel (manufactured by Sanofi Pasteur MSD and distributed by Movianto UK Ltd), in October 2004 [8]. From June 2014, the 3-component aP, Infanrix IPV Hib (manufactured by GlaxoSmithKline and distributed by Movianto UK Ltd) [8], was used in the national programme and both this and Pediacel were available in England until a recommendation to introduce hepatitis B into the routine programme. For babies born from 1 August 2017, a hexavalent product is in use (DTaP/IPV/Hib/HepB, Infanrix hexa).

It is well recognised that not all pertussis vaccines are the same. Differences in efficacy and effectiveness have been demonstrated between the licensed wP vaccines as well as between aP vaccines [9]. In 2008, it was agreed by the UK Joint Committee on Vaccination and Immunisation (JCVI) that only aP vaccines with three or more components should be used for the national immunisation programme as vaccines with one or two components were likely to be less effective [8,10]. Furthermore, an efficacy study presented at the 2014 World Health Organization Strategic Advisory Group of Experts (WHO SAGE) Working Groups on pertussis vaccines meeting compared the efficacy of multiple component aP vaccines against a UK wP vaccine [11,12]. The results indicated that the efficacy of the 5-component aP primary vaccine was not statistically different compared to efficacy for wP against culture-confirmed pertussis. However, the efficacy of aP vaccines against mild disease was dependent on the number of components in the vaccine [11].

The aim of the 2001 preschool booster programme was to increase herd immunity and reduce the transmission of pertussis to young infants, given the evidence that older siblings in the household were an important source of infection for these infants [13]. An economic evaluation of an aP booster programme demonstrated it to be a cost-effective intervention in the UK [14] and given the high reactogenicity of wP after a primary course, an aP booster was introduced. The pertussis preschool booster vaccine was first introduced in England using a 3-component acellular booster pertussis vaccination (Td3aP-IPV, Infanrix-IPV) [15]. In 2001, a study estimated that over a 5-year period from the introduction of the pertussis booster vaccine, it prevented a total of 1,400 pertussis cases in the UK [16]. From August 2004, a 5- component booster aP, Td5aP-IPV/Repevax, was also made available and GPs were able to order and offer it either as part of the preschool booster programme. To date, there is no evidence of any significant difference in protection between these two booster vaccines.

In England, a national pertussis outbreak was declared in 2012 which led to the introduction of the maternal vaccination programme [17]. Since then, the number of confirmed pertussis cases continues to exceed levels seen before the 2012 outbreak peak [17] and pertussis outbreaks have arisen in secondary schools (students ages 11–16 years), probably reflecting waning immunity [18].

## Outbreak detection

In early March 2019, a single pertussis case was confirmed (with a serology test performed at a hospital laboratory using a commercial kit) in a junior school (students in school years 3–6, ages 7–11 years) in South East England. The index case had a symptom onset date in late February and their sibling, attending the infant school (ages 4–7 years), also tested positive for pertussis.

The local Public Health England (PHE) Health Protection Team (HPT) was notified of the pertussis case in the junior school. In response to the initial case, a letter was sent in early March to the parents of the students attending the junior school raising awareness of pertussis, which was followed by several reports of students at the school being absent with coughs. Within the same week of sending the letter, four additional students at the school were notified as possible cases with onset dates varying between late January 2019 and late February 2019, 26 days apart. By end of April 2019, 18 students had presented to their GP with clinical symptoms and were tested for pertussis by the GP. As a result, an incident management team meeting was convened, as this was the first pertussis outbreak in a junior or primary school notified to PHE since the introduction of the preschool booster. It was agreed to offer oral fluids tests to all students in the junior school and undertake an extensive investigation to better understand the reasons for this outbreak and the potential implications for the wider school-aged population in the UK. Of note, most students had received the same vaccine product for their primary series although the preschool booster vaccine product varied.

The aim of this analysis was to evaluate the extent of the pertussis outbreak in a junior school in South East England and determine whether the odds of pertussis among the students in this outbreak varied with the two preschool booster vaccines.

## Methods

In light of an increasing number of pertussis cases in the school, a decision was taken to conduct enhanced case finding by asking parents of all students in the junior school to complete a clinical questionnaire and consenting for their child to have an oral fluid sample taken, to better understand the extent of transmission. The oral fluid assay determines the pertussis toxin IgG titre which when raised can be used as a marker of recent pertussis infection [19].

The clinical questionnaire asked about onset and duration of any symptoms and number of days absent from school. Detailed information on the pertussis-containing vaccines received, including vaccination date, manufacturer/batch number for the primary series and booster vaccines, were requested for each child from the GP practice where they were registered.

### Oral fluid testing 

Oral fluid samples were offered to all students in the junior school and were collected by the local HPT and sent to the pertussis National Reference Laboratory to test for IgG antibodies against pertussis toxin (anti-PT) at PHE’s National Infection Service in Colindale, London. Oral fluid testing is offered as part of PHE’s national surveillance of notified pertussis cases in 2–16-year-olds [20]. In the literature, patients who have at least a 2-week history of cough, in the absence of recent vaccination with a pertussis-containing vaccine, and a titre of > 70 aU (arbitrary units of anti-*B. pertussis* toxin IgG antibody) – equivalent to a serum threshold > 70 IU/mL (international units) of anti-PT IgG titre – are reported as consistent with recent pertussis infection; a titre of 60–70 aU is reported as elevated close to the diagnostic threshold for a single sample and a titre < 60 aU as no evidence of recent pertussis infection [20,21]. However, given that these samples in this situation were taken as part of an outbreak investigation for asymptomatic and symptomatic individuals, samples with PT IgG titres of 60–70 aU were also considered to be consistent with recent pertussis infection to take account of those only recently exposed who may be mounting a response.

### Case definitions 

We conducted a case–control study assessing the students in the junior school at the time of the pertussis outbreak. Controls were students attending the junior school in South East England who did not have elevated serum anti-PT IgG titre, positive PCR IS*481* or IS*481* and *ptxP* detected, or oral fluid anti-PT IgG titres. Cases attending the junior school were defined as:

- Symptomatic with a clinically compatible illness and an oral fluid anti-PT IgG titre of ≥ 60 aU, positive PCR (IS*481*, or IS*481* and *ptxP* detected [22]), or serum sample (anti-PT IgG titre of > 70 IU/mL);
- Asymptomatic with a high or elevated oral fluid anti-PT IgG titre ≥ 60 aU;
- Symptomatic with a clinically compatible illness and treated with antibiotics without laboratory evidence of pertussis (based on a review by a clinician at PHE with vaccination status blinded) to clinically agree that the cases had pertussis.

### Statistical methods

Students were included in the analysis if they met the following criteria based on the routine immunisation schedule [7]:

- First dose of pertussis vaccine from 42 days-old (6 weeks);
- All three primary doses administered by the time the infant turned 1 year;
- All three primary doses were offered a minimum of 28 days apart, but one interval could be a minimum of 21 days (3 weeks);
- The booster dose was a minimum of 3 years after the final primary dose;
- The booster vaccine was no later than the child’s fifth birthday.

We assessed the total number of students at the junior school and distribution of pertussis cases by sex and school year. We used univariable logistic regression and multivariable logistic regression adjusting for vaccine, type, school year and sex to assess whether there was an association between Repevax, Infanrix-IPV, no booster vaccine and unknown vaccine on all confirmed cases in this outbreak (clinical and laboratory confirmed). Statistically significant variables (p ≤ 0.05 and confidence intervals which did not include the value zero) were added to the multivariable model. A test for interaction of school year and vaccine type was completed to check for a linear effect as a proxy for time since booster vaccination (possible differential speed of waning between the vaccines). The model and test for interaction was rerun as a sensitivity analysis without the asymptomatic cases for comparison. 

We conducted a secondary logistic regression analysis (adjusting for the same variables in the initial analyses) including the students that had received their booster dose early (less than 3 years before their final dose) and later (after age 5 years) but still met the inclusion criteria based on the routine immunisation schedule. All analyses were conducted in Stata 15 (StataCorp LLC).

### Ethical statement

PHE has legal permission, provided by Regulation 3 of The Health Service (Control of Patient Information) Regulations 2002, to process patient confidential information for national surveillance of communicable diseases (http://www.legislation.gov.uk/uksi/2002/1438/regulation/3/made). This includes PHE’s responsibility to monitor the safety and effectiveness of vaccines. Individual patient consent is therefore not required by PHE for pertussis cases.

## Results

**Figure 1:**
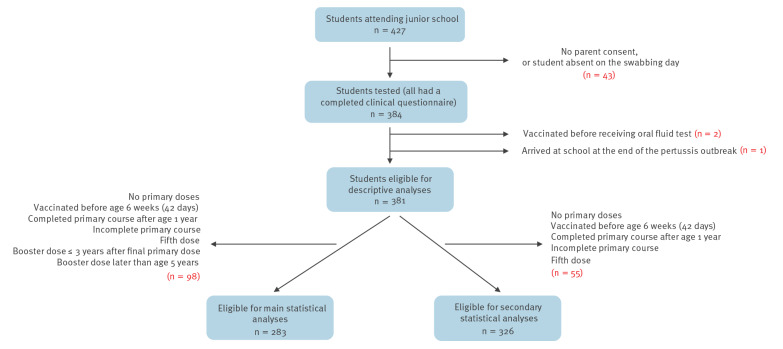
Inclusion criteria, pertussis outbreak investigation, junior school, South East England, 2019 (n = 427)

A total of 427 students attended the junior school at the time of the outbreak. Of these 427 students, 384 completed the clinical questionnaire, had an oral fluid test, and vaccination history was obtained from their GP records (Figure 1). 

Oral fluid tests were offered at the school on 1 and 2 May 2019. Of the 384 students, 379 had an oral fluid test during the school swabbing. A total of 18 students were tested at their GP prior to the school swabbing, where 11 were laboratory-confirmed for pertussis with high or elevated anti-PT IgG titres or IS*481*, or IS*481* and *ptxP* detected (one by serology, 10 by PCR). Thirteen of these 18 students were retested in the school. Of the 13, nine retested positive at the school swabbing, while four tested positive at the school after previously testing negative at the GP. The remaining five students tested positive at their GP prior to the school swabbing and chose not retest in the school. 

Three of the 384 students with complete data were excluded from the analysis (one had joined the school a week before the immunisation campaign and two students had been vaccinated within a week before the collection of oral fluid), leaving 381 students for the analyses. A total of 134 of 381 (35.2%) students at the school were classified as pertussis cases during the outbreak (133 based on oral fluid testing and one clinically diagnosed). Thirty-nine (29.1%) of the confirmed cases were asymptomatic and did not report any coughing. 

### Vaccination history

All students attending the junior school were born before the introduction of maternal pertussis immunisation in England (which began in October 2012). In addition, all students attending the junior school were born between 2008 and 2012 and during that period, all infants received Pediacel vaccine for the primary series, as this was the only vaccine offered at the time. Students were offered Repevax or Infanrix-IPV for their booster dose (Table 1).

**Table 1:** Vaccine types used for routine pertussis immunisation of age cohorts included in the outbreak investigation, England

School year (age in years)	Birth year (September to August)	Years received infant vaccine	Vaccine types that year	Years received booster vaccine	Vaccine types that year
3 (7–8)	2011/12	2011/13	Pediacel	2015/16	Repevax or Infanrix-IPV
4 (8–9)	2010/11	2010/12		2014/15	
5 (9–10)	2009/10	2009/11		2013/14	
6 (10 -11)	2008/09	2008/10		2012/13	

Students who had been immunised according to the routine immunisation schedule were included in the statistical analyses. We excluded 98 of 381 (25.7%) students from the outbreak investigation who did not meet the investigation criteria because they had an incomplete immunisation schedule, additional doses, or the doses did not adhere to the schedule (Figure 1). Of 381 students, 42 (11.0%) had an incomplete or no primary vaccination and 47 (12.3%) had received the preschool booster after their fifth birthday or had not had a booster dose while the remaining nine (2.4%) had a combination of incomplete primary and booster vaccinations. For the secondary statistical analyses, students that received the booster dose early and late were included in the model (Figure 1). 

### Descriptive analyses

**Figure 2:**
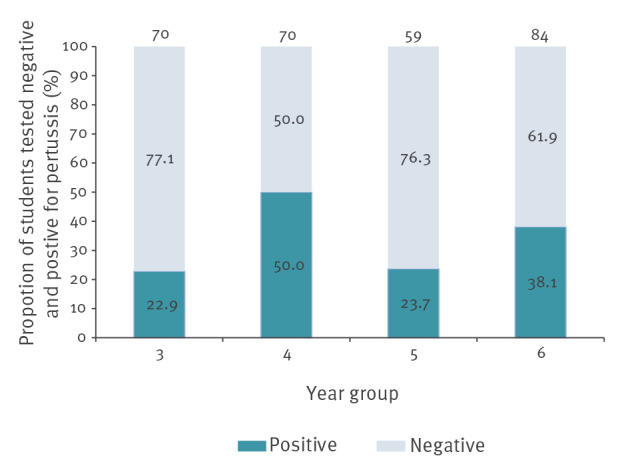
Students with a completed immunisation course who tested positive and negative for pertussis, junior school outbreak, South East England, 2019 (n = 283)

The index case of the pertussis outbreak was a female student. Among the students that met the criteria based on the routine immunisation schedule, the total number of boys and girls in the school was approximately even (145 and 138, respectively among the 283 included students). However, 56 (40.6%) girls were positive for pertussis as opposed to 41 (28.3%) boys. The number of students in each year group was also evenly distributed, with a higher proportion of positive pertussis cases in school year 4, the year group where the first case was confirmed (Figure 2).

Positive pertussis cases were defined by oral fluid test result ≥ 60 aU, (arbitrary units of anti-*Bordetella pertussis* toxin IgG) or a serum test result with > 70 IU anti-B*. pertussis* toxin IgG or with PCR detection of insertion element *IS481* or *IS481* and pertussis toxin promoter region (*ptxP*).

A total of 186 of 283 (65.7%) students had no laboratory evidence of pertussis infection, while 96 (33.9%) tested positive for pertussis and one (0.4%) was clinically diagnosed with pertussis. Of those who tested positive, 26 were asymptomatic. 

Of the two booster vaccines offered to the students before school entry, Repevax was offered to more children than Infanrix-IPV, 167 vs 86, respectively. Eight students did not have a booster vaccine and 22 had unknown vaccination status. 

### Statistical analyses

A total of 283 students were included in the main logistic regression analyses. The multivariable logistic regression, adjusted for vaccine type, school year and sex, indicated no statistically significant difference between Infanrix-IPV and Repevax vaccines. Compared with year 3 (reference age group), the odds of pertussis were significantly increased for children in school year 4 (odds ratio (OR) = 3.86; 95% CI: 1.80–8.30) and in year 6 (OR = 2.45; 95% CI: 1.08–5.53), but there was no significant difference in year 5 (Table 2 and Figure 2). The primary case was identified in a year 4 student. In addition, the odds of pertussis were significantly lower among boys (OR = 0.51; 95% CI: 0.30–0.86) compared with girls (Table 2). In a test for interaction of school year and preschool booster vaccine type, there was no linear trend in the odds of having pertussis by school year or type of preschool booster vaccine (OR = 0.43; 95% CI: 0.17–1.06)). The exclusion of asymptomatic cases did not change the overall findings.

**Table 2:** Students with/without laboratory evidence of pertussis and multivariable logistic regression analysis, South East England junior school, 2019 (n = 283)

Characteristic		Number of students (n = 283)	Number with evidence of pertussis infection (n = 97)	Crude OR (95% CI)	AOR (95% CI)	p value for difference across all levels
Booster vaccine type	Infanrix-IPV	86	29	Reference		
	Repevax	167	54	0.94 (0.54–1.63)	0.72 (0.38–1.39)	0.40
	Unvaccinated	8	4	1.97 (0.46–8.43)	1.55 (0.34–7.07)	
	Not known	22	10	1.64 (0.63–4.24)	1.38 (0.49–3.86)	
School year	3	70	16	Reference		
	4	70	35	3.37 (1.63–6.99)	3.86 (1.80–8.30)	0.001
	5	59	14	1.05 (0.46–2.38)	1.23 (0.52–2.92)	
	6	84	32	2.08 (1.02–4.23)	2.45 (1.08–5.53)	
Sex	Female	138	56	Reference		
	Male	145	41	0.58 (0.35–0.95)	0.51 (0.30–0.86)	0.011

AOR: adjusted odds ratio; CI: confidence interval; OR: odds ratio.

The secondary logistic regression analysis comprised 326 students, including children who had received the booster dose early (less than 3 years after their final primary dose) or late (later than age 5 years). There was less difference in the odds of pertussis between the two products and the OR remained insignificant (OR for Repevax changed to 0.83 (95% CI: 0.45–1.51) compared with the primary logistic regression analysis. A test for interaction between school year and vaccine type indicated no evidence of differential waning in this outbreak investigation (p = 0.095).

## Outbreak control measures

A total of 427 students in school years 3–6 were enrolled at the junior school at the time of the outbreak. In response to the outbreak, all students in the school were offered a booster dose of pertussis vaccine and 363 of 427 students (85%) were vaccinated. No students were hospitalised with pertussis and only one pertussis case was reported 9 months after the outbreak.

## Discussion

Pertussis activity has remained at heightened levels across England since 2012 and while infant disease has been at low levels following the introduction of the maternal programme, rates of disease in school-age children appear to be increasing [17]. In this outbreak investigation, we present the results from an investigation of a pertussis outbreak identified in a junior school In England. 

Early accurate diagnosis is important to control transmission of pertussis [13]. Most pertussis infections in adults and adolescents are asymptomatic or oligosymptomatic [13], therefore the disease can spread easily as people may not be aware that they have pertussis and may not undergo confirmatory testing. Furthermore, an untreated pertussis case can lead to 11 to 15 secondary cases in a non-immune population and the control of pertussis is more difficult when a vaccine may not induce lasting immunity [23]. 

In this outbreak, a total of 39 students (of whom 26 were included in the statistical analyses) with laboratory evidence of recent pertussis infection were not recorded as having a cough. Given the nature of the study, where parents completed the questionnaire retrospectively in late April 2019 (based on their child’s symptoms since January 2019), there was a risk of recall bias. However, it is also possible that these students were protected from vaccine doses that they had received earlier before the outbreak. The sensitivity analysis indicated that even when excluding those without reported symptoms, there remained no difference in the risk of pertussis among those who received either the 3 or 5 component vaccine as their preschool booster. It is worth noting that all symptomatic cases linked to this outbreak reported mild symptoms with no hospitalisations. One further pertussis case (confirmed by oral fluid test) occurred in the junior school 9 months later; this case is not included in this study.

This outbreak provided a unique opportunity to evaluate the comparative protection afforded by the two available products for the preschool booster programme. The two vaccines differ in two pertussis antigenic components and in the amount of toxoid or antigen in some of the shared components. Infanrix-IPV is licensed for both primary and booster immunisation, whereas Repevax is licensed as a booster following primary immunisation. Of the three shared pertussis antigens, Repevax has greater amounts of all three with 10 × more pertussis toxid than Infanrix (25 vs 2.5 µg). Our study did not see a difference in the odds of pertussis between the two different preschool booster vaccines offered in the UK (Infanrix-IPV and Repevax). The OR of pertussis was higher among girls, which may be due to the social interactions of the index case, who was a girl.

The routine immunisation schedule recommends that children receive their booster dose at the age of 3 years and 4 months. A total of 12.3% of the students at the junior school received the preschool booster after their fifth birthday or had no booster dose at all. This is similar to the national DTaP booster coverage at 5 years: In the period 2013 to 2016 when the students in this outbreak investigation would have been eligible for the preschool booster vaccine, 11.1–13.7% of UK children were unvaccinated [24]. Information on vaccine manufacturer and batch number may be missing when students change to a GP practice with a different system supplier and coding language from their previous GP. Students who moved to the local area from another country may have incomplete vaccination histories and may have received different vaccines with different timing according to immunisation schedules abroad. The proportion of students with incomplete or no primary vaccines at the junior school (11.0%) was greater than the national proportion of unvaccinated children which ranged from 4.2% to 4.4% of children who had not completed their primary course of DTaP/IPV/Hib by their fifth birthday between 2012 and 2017 [24]. The high proportion of unvaccinated/partially vaccinated students compared with the national coverage and the high proportion of students not adhering to the recommended schedule is likely to have contributed to transmission within the school population. Conversely, coverage could have been higher if the 64 students for whom we could not obtain GP records had received a pertussis vaccine; among those students with accessible GP records, three had notes of being vaccinated outside of England but the record did not specify vaccine type. Research suggests that it is important which priming vaccine is used in order to achieve a robust immune response and reduce the transmission of pertussis [25]. A study in Australia assessed children born during the transition from wP to aP and showed that children who received aP had higher rates of pertussis than those vaccinated with wP; among those who received a mixed course, pertussis rates were higher when aP was the first dose [26]. Furthermore, a study in England suggested that priming with a 5-component aP vaccine may have an effect more similar to wP than a 3-component aP vaccine [27]. In our outbreak investigation, all students were eligible for the 5-component aP primary vaccine as the 3-component aP vaccine was not offered throughout England, therefore it was not possible to compare the different primary vaccines offered in England. However, in our investigation there appeared to be no significant difference between the two preschool booster vaccines used in England.
