# Correction for Euro Surveill. 2021;26(12)

**DOI:** 10.2807/1560-7917.ES.2022.27.2.220113c

**Published:** 2022-01-13

**Authors:** Eurosurveillance editorial team

**Affiliations:** 1 European Centre for Disease Prevention and Control (ECDC), Stockholm, Sweden

In the article ‘Investigation of a pertussis outbreak and comparison of two acellular booster pertussis vaccines in a junior school in South East England, 2019’ by Tessier et al., published on 25 March 2021, Repevax was mistakenly stated as having ten times more pertussis toxid than Infanrix. The text was corrected on 13 January 2022 on request of the authors. 

